# Brain-derived neurotrophic factor prevents LPS-induced dysregulation of GABAergic interneuron markers in mouse hippocampus

**DOI:** 10.3389/fncel.2025.1675003

**Published:** 2025-09-25

**Authors:** Sara Rezaei, Mounira Banasr, Thomas D. Prevot, Yashika Bansal, Erica Vieira, Etienne Sibille

**Affiliations:** 1 Centre for Addiction and Mental Health (CAMH), Toronto, ON, Canada; 2 Department of Pharmacology and Toxicology, University of Toronto, Toronto, ON, Canada; 3 Department of Psychiatry, University of Toronto, Toronto, ON, Canada

**Keywords:** brain-derived neurotrophic factor, lipopolysaccharide, GABAergic interneurons, inflammation, neuropsychiatric disorders

## Abstract

**Background:**

Inflammation causes reduced markers of GABAergic interneurons and brain-derived neurotrophic factor (BDNF) in the hippocampus, features often associated with neuropsychiatric disease pathophysiology. However, the mechanism connecting inflammation to GABAergic markers remains unclear. We hypothesized that reduced BDNF mediates the effects of LPS on GABAergic markers and that hippocampal BDNF infusion would prevent LPS-induced reduction in somatostatin (SST), and coexpressed markers, including cortistatin (CORT), and neuropeptide Y (NPY).

**Method:**

C57BL/6 mice (*n* = 14; 12–14 weeks old; 50% female) received intracerebral administration of BDNF (250 ng) or vehicle control in the hippocampus via stereotaxic surgery (unilateral). Thirty minutes after BDNF administration, intraperitoneal injection of LPS (2 mg/kg) or phosphate buffered saline (PBS) was performed and mice were euthanized 18 h post LPS-injection. The hippocampus was collected for investigation of cellular markers using quantitative PCR and enzyme-linked immunosorbent assay (ELISA).

**Results:**

LPS administration in mice that did not receive pre-treatment with BDNF led to a significant reduction in *mRNA* levels of *Bdnf* (*p* = 0.0049), *Sst* (*p* = 0.0416), *Npy* (*p* = 0.0088), and *Cort* (*p* = 0.0055). BDNF infusion into the hippocampus prior to LPS injection prevented the reduction in *Bdnf, Sst,* and *Cort m*RNA expression. BDNF also prevented the LPS-induced effect on protein levels of BDNF, SST and NPY. BDNF prevention of LPS effects occurred in the context of sustained elevation of inflammatory markers (interleukin 1-beta and glial fibrillary acidic protein).

**Conclusion:**

BDNF may protect SST GABAergic interneurons from LPS-induced inflammation, providing novel insights into the molecular mechanisms linking inflammation and GABAergic dysfunction in neuropsychiatric diseases.

## Introduction

The underlying pathophysiology of neuropsychiatric disorders involves increased inflammation (Miller et al., 2009), disruption of the inhibitory gamma-aminobutyric acidergic (GABAergic) neurotransmitter system (Newton et al., 2019), and reduced neuroplasticity (Tripp et al., 2012). Understanding the link between these biological changes can facilitate improved therapeutics.

The GABAergic system comprises multiple types of interneurons: somatostatin (SST), parvalbumin (PV) and vasoactive intestinal peptide (VIP)-expressing interneurons. Extended set of markers co-expressed with these main interneuron subtypes include neuropeptide Y (NPY), cortistatin (CORT), cholecystokinin (CCK) and corticotropin-releasing hormone corticotropin-releasing hormone (CRH) (Tremblay et al., 2016). Interneurons release GABA and innervate glutamatergic pyramidal neurons, forming cell microcircuits that regulate the balance between excitation and inhibition, thereby contributing to neuronal information processing (Fee et al., 2017). Clinical and postmortem studies have shown reduced GABA levels (Newton et al., 2019) and markers of GABAergic interneurons, including *Sst* and genes co-expressed with SST cells, such as *Cort* and *Npy* across neuropsychiatric diseases including major depressive disorder (MDD; Guilloux et al., 2012; Sibille et al., 2011; Tripp et al., 2011), schizophrenia (Mellios et al., 2009; Guillozet-Bongaarts et al., 2014; Volk et al., 2012; Dienel et al., 2025), and bipolar disease (Sibille et al., 2011; Konradi et al., 2004).

A proposed mechanism underlying the reduction of GABAergic interneurons is reduced neurotrophic support, particularly from brain-derived neurotrophic factor (BDNF), which mediates neuronal plasticity and survival (Duman et al., 1997). Studies report reduced BDNF levels in cortical areas, plasma and hippocampus (HPC) of patients with MDD (Duman and Monteggia, 2006; Dwivedi et al., 2003; Karege et al., 2005) and schizophrenia (Green et al., 2011; Durany et al., 2001).

Parallel to the GABAergic and neurotrophic deficits, studies have shown increased levels of inflammatory markers in individuals with MDD (Howren et al., 2009; Dowlati et al., 2010; Hiles et al., 2012). Microglial activation is seen in patients with MDD (Setiawan et al., 2015), schizophrenia (van Berckel et al., 2008), and bipolar I disorder (Haarman et al., 2014). Astrocyte dysfunction has also been reported in post-mortem MDD and post-traumatic stress disorder patients (Bansal et al., 2024). Recently, we showed that lipopolysaccharide (LPS)-induced inflammation induces deficits in GABAergic interneuron markers *Sst, Cort, Npy,* and *Cck* in the prefrontal cortex (PFC) and HPC of mice, and a reduction in *Bdnf* expression (Rezaei et al., 2024). The mechanism by which inflammation affects the GABAergic interneurons remains unclear, but a positive correlation between the expression of markers of these two systems suggests BDNF as a putative regulator (Rezaei et al., 2024).

In this study, we investigated the interplay between inflammation, BDNF and GABA neuron dysfunction. We used LPS, a cell wall component of gram-negative bacteria, to induce a peripheral immune response that propagates into the brain (Dantzer et al., 2008). We infused BDNF mature protein into the HPC of mice and investigated the expression levels of GABAergic interneuron markers *Sst*, *Npy*, *Cort*, *Crh*, *Vip*, and *Cck* following 18 h of LPS exposure (allowing changes in GABAergic markers, as in Rezaei et al., 2024). Given the role of microglia and astrocytes in mediating neuroinflammatory responses (Farina et al., 2007; Wohleb, 2016), contributing to BDNF synthesis (Albini et al., 2023; Parkhurst et al., 2013), and their potential to act as downstream targets of tropomyosin receptor kinase B (TrkB)-dependent trophic pathways (Wu et al., 2020), we measured ionized calcium binding adaptor molecule 1 (*Iba1*) as a marker of microglia activation, glial fibrillary acidic protein *(Gfap)* as a marker of astrocyte activation, and IL-1β as a key pro-inflammatory cytokine to assess the inflammatory response to LPS and BDNF treatment.

Based on a hypothesized role of BDNF in mediating effects of LPS on GABAergic markers, we predicted that BDNF pretreatment would prevent the LPS-induced deficits of GABAergic interneuron markers in the hippocampus.

## Materials and methods

### Animals

Male and female C57BL/6 mice (*n* = 14, 4-5/group, 12–14 weeks old, 50% female; Jackson Laboratories, Bar Harbor, ME) were housed in groups of four within individually ventilated cages (IVC) under a 12-h light/dark cycle with ad libitum access to food and water. Mice were habituated to the facility for 2 weeks and handled for 3 days to minimize stress (Marcotte et al., 2021).

All procedures complied with the Canadian Council on Animal Care guidelines and were approved by the Animal Care Committee at Center for Addiction and Mental Health (CAMH).

### Surgery, drug infusion and brain dissection

Mice were anesthetized with isoflurane and guide cannulae (Protech International INC. Boerne, TX) were stereostaxically placed into the HPC (AP: −1.8 mm; ML:+/−0.4 mm; DV: −1.8). Cannula implantation was unilateral and inserted on either the left or right side in a balanced manner across the groups. Following one-week of recovery, mice received intracranial infusion of 0.5 μL recombinant human BDNF (R&D systems, Biotechne, Minneapolis) at a concentration of 0.5 μg/μL (a total 250 ng/animal) or sterile PBS at a rate of 0.1ul/min. Thirty (30) minutes later, intraperitoneal injection of ultra-pure LPS (2 mg/kg, InvivoGen, San Diego, CA) or PBS was performed. We selected 30 min because studies have shown increased BDNF immunolabeling 30 min after infusion and peaking at 2 h (Shirayama et al., 2002). The groups of mice were PBS/PBS, BDNF/PBS, PBS/LPS, BDNF/LPS. After 18 h, mice were euthanized by rapid cervical dislocation, and whole hippocampal tissue (dorsal and ventral) was collected. Detailed surgical procedures and infusion protocols are provided in SI Section 1.

### Quantitative real-time PCR and enzyme-linked immunosorbent assay (ELISA)

Hippocampal RNA was extracted using the Allprep RNA/protein kit and cDNA was synthesized with SuperScript VILO cDNA Synthesis Kit. qPCR was performed using SYBR Green supermix with primers listed in Supplementary Table 1. Hippocampal protein was quantified to measure the protein levels of the neuropeptides using ELISA kits for SST, NPY, CORT, IL-1β and BDNF. Relative gene expression was calculated using the 2^(-dCt) method. A complete description of RNA, protein extraction, cDNA synthesis, qPCR analysis, and ELISA procedures are provided in SI Section 1.

### Statistical analysis

Statistical analysis was conducted using GraphPad Prism 10. Data from both sexes were combined. Our analysis focused on pre-specified biologically-driven comparisons, using unpaired *t*-tests. We conducted two planned comparisons (1): PBS/PBS vs. PBS/LPS to confirm the LPS-induced deficit, and (2) PBS/LPS vs. BDNF/LPS to test for the protective effect of BDNF.

## Results

### BDNF infusion prevented LPS-induced reduction in *Bdnf*, *Sst*, *Cort*, and *Npy* mRNA, with no effect on, *Pv*, *Vip*, *Crh* or *Cck*

**Figure 1:**
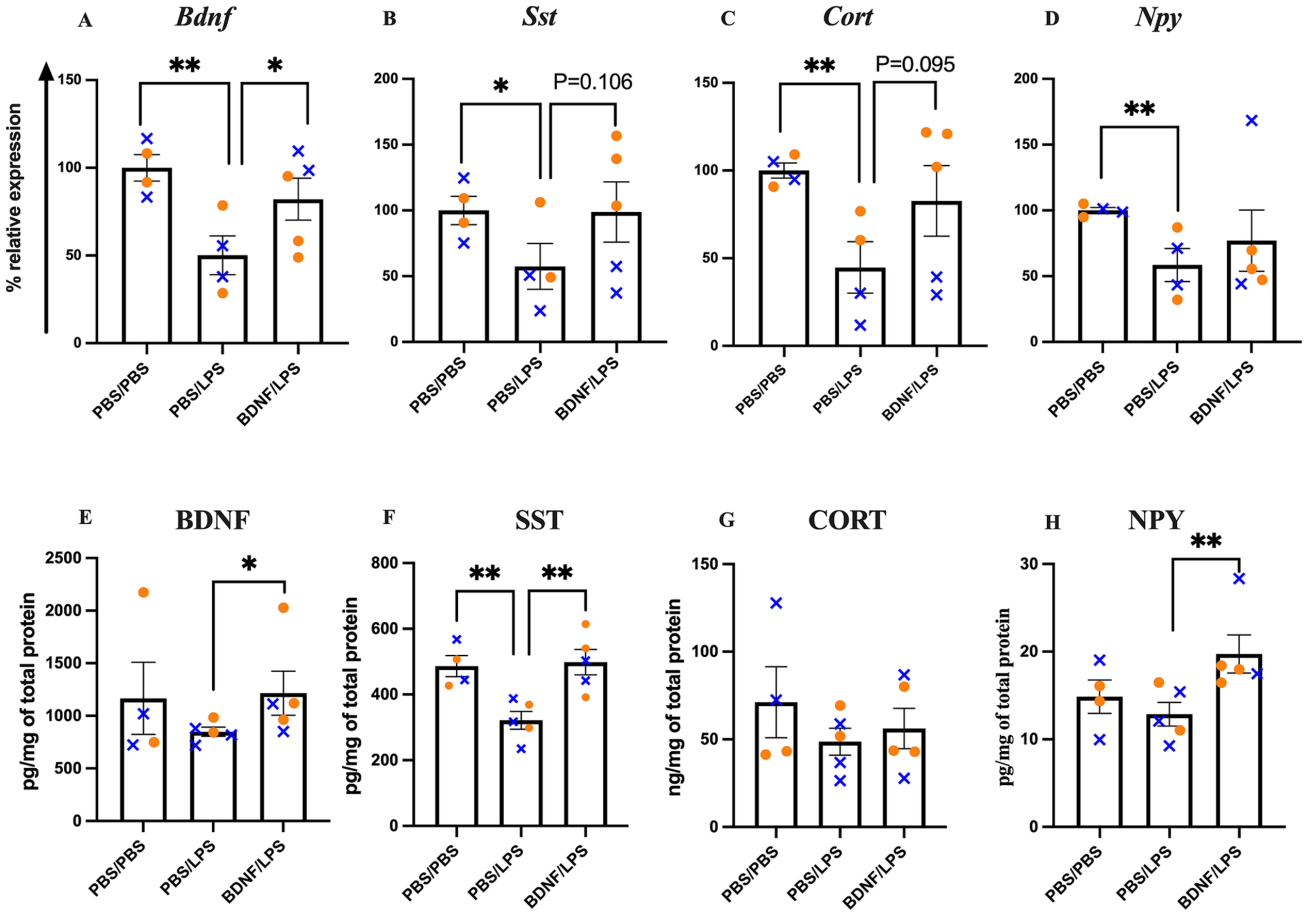
Effect of LPS and BDNF on hippocampal *Bdnf*, *Sst*, *Cort*, and *Npy* mRNA and protein levels. **(A–D)** *Bdnf*, *Sst*, *Cort*, and *Npy* mRNA expression levels. LPS significantly reduced expression of all four genes compared to PBS controls. BDNF infusion fully blocked the reduction in *Bdnf* **(A)**, partially blocked reductions in *Sst* **(B)** and *Cort* **(C)**, and modestly increased *Npy* **(D)**. **(E–H)** Corresponding protein levels for BDNF, SST, CORT, and NPY. LPS had no significant effect on BDNF **(E)**, CORT **(G)**, or NPY **(H)** protein levels, but significantly reduced SST protein levels **(F)**. BDNF infusion significantly increased BDNF **(E)**, SST **(F)**, and NPY **(H)** protein levels compared to PBS/LPS, but had no effect on CORT **(G)** protein levels. Results are expressed as individual mice and mean ± SEM (*n* = 4-5/group; 50% female). Females are shown as orange circles and males as blue x symbol. *p < 0.05 and **p < 0.01.

Eighteen hours after LPS exposure, hippocampal *Bdnf* expression was significantly reduced. Two by two comparisons revealed a significant decrease in PBS/LPS group compared to PBS/PBS group (*p* = 0.0049). BDNF infusion fully blocked the effect of LPS, as *Bdnf* expression increased in the BDNF/LPS group compared to PBS/LPS (*p* = 0.0482; Figure 1A).

For *Sst*, there was a significant decrease in the PBS/LPS group compared to PBS/PBS group (*p* = 0.0416), while BDNF partially blocked this effect, resulting in a 72% increase in *Sst* levels in the BDNF/LPS group compared to the PBS/LPS group (*p* = 0.1062; Figure 1B).

LPS significantly decreased *Cort* expression, with a reduction observed in the PBS/LPS group compared to PBS/PBS group (*p* = 0.0055). BDNF partially blocked the effect of LPS on *Cort* as evidenced by an 84% increase in *Cort* expression in the BDNF/LPS group compared to PBS/LPS (*p* = 0.095; Figure 1C).

LPS significantly decreased *Npy* expression, with a reduction in PBS/LPS group compared to PBS/PBS group (*p* = 0.0088). BDNF partially blocked the effect of LPS on *Npy* as evidenced by the 31% increase in *Npy* expression in the BDNF/LPS group compared to PBS/LPS (*p* = 0.2692; Figure 1D). In the BDNF/PBS group there was a significant increase only in *Npy* mRNA expression compared to PBS/PBS group (*p* = 0.0445; Supplementary Figure 2C).

Results for *Pv, Vip, Crh* and *Cck* are in Supplementary Figure 1.

### BDNF infusion prevented LPS-induced reduction in SST, NPY, and BDNF protein levels, but had no effect on CORT

There was no effect of LPS on BDNF protein levels as two by two comparisons showed no change in BDNF levels in the PBS/LPS group compared to PBS/PBS group (*p* = 0.1660). There was a significant increase in BDNF levels in the BDNF/LPS group (*p* = 0.0278) compared to PBS/LPS (Figure 1E).

LPS significantly decreased SST protein levels in PBS/LPS compared to PBS/PBS group (*p* = 0.0055). BDNF fully blocked this effect, as SST protein levels increased by 55% in the BDNF/LPS group compared to PBS/LPS (*p* = 0.0056; Figure 1F).

There was no significant effect of LPS on CORT protein levels, as CORT levels did not differ between PBS/LPS group compared to PBS/PBS group (*p* = 0.1456), nor between BDNF/LPS group and PBS/LPS group (*p* = 0.5971; Figure 1G).

There was no effect of LPS on NPY protein levels, as NPY levels did not differ between the PBS/LPS group compared to PBS/PBS group (*p* = 0.2027). However, NPY levels were significantly increased in the BDNF/LPS group compared to PBS/LPS (*p* = 0.0079; Figure 1H).

### LPS increased IL1-beta protein levels and *Gfap* mRNA levels and decreased *Iba1*

**Figure 2:**
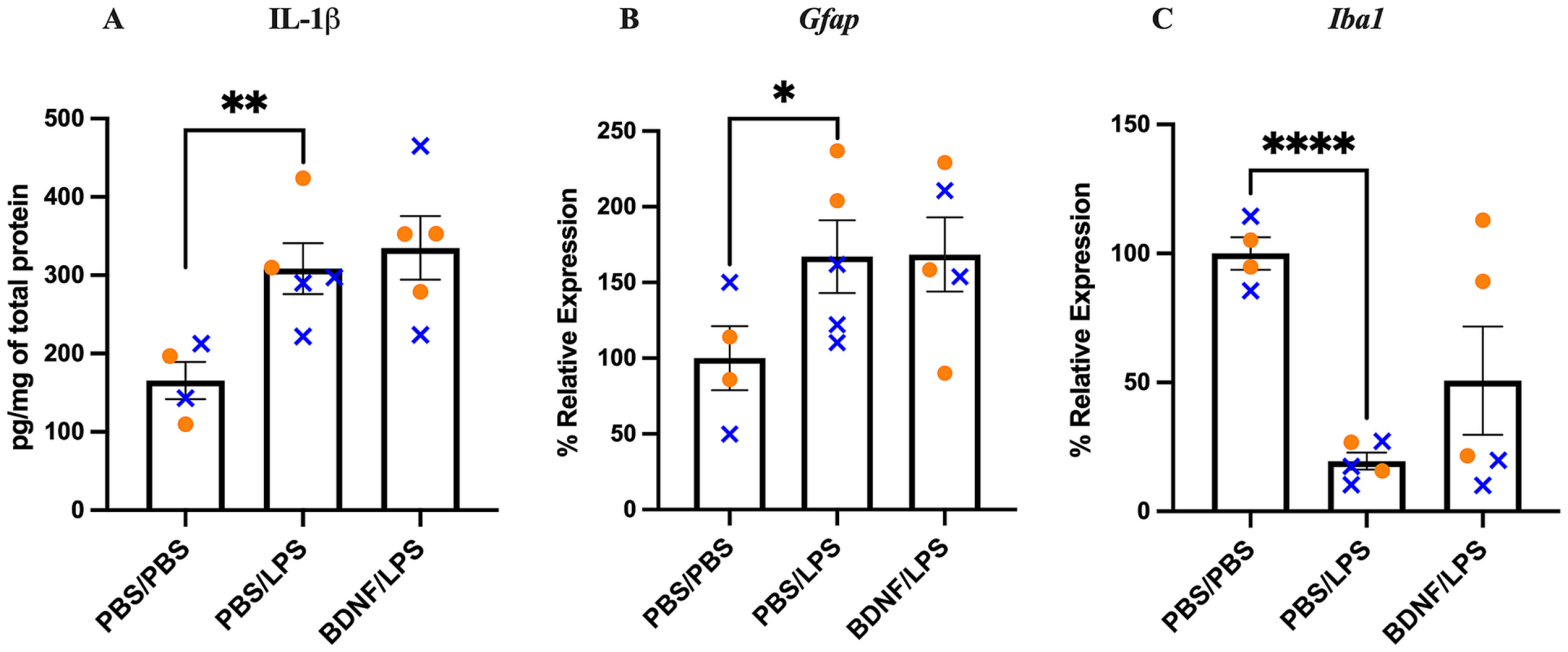
Effect of LPS and BDNF on hippocampal IL-1beta protein, *Gfap* and *Iba1* mRNA levels. **(A)** LPS significantly increased IL-1beta protein levels compared to PBS controls. BDNF infusion did not significantly alter IL-1beta levels compared to the PBS/LPS group. **(B)** LPS significantly increased *Gfap* mRNA expression, while BDNF infusion had no effect on LPS-induced *Gfap* expression. **(C)** LPS significantly decreased *Iba1* mRNA expression. BDNF had no significant effect on *Iba1* expression in the BDNF/LPS group compared to PBS/LPS group. Results are expressed as individual animals and mean ± SEM (*n* = 4-5/group; 50% female). Females are shown as orange circles and males as blue x symbol. *p < 0.05, **p < 0.01, and ****p < 0.0001.

LPS significantly increased IL-1β protein levels in the PBS/LPS group compared to PBS/PBS group (*p* = 0.006; Figure 2A). There was no significant change in BDNF/LPS group compared to PBS/LPS group (*p* = 0.314).

LPS significantly increased *Gfap* in the PBS/LPS group compared to PBS/PBS group (*p* = 0.0408). There was no significant change in BDNF/LPS group compared to PBS/LPS group (*p* = 0.480; Figure 2B).

LPS significantly decreased *Iba1* expression in the PBS/LPS group compared to PBS/PBS group (*p* < 0.0001). There was a trend of increase in *Iba1* in the BDNF/LPS group compared to PBS/LPS group (*p* = 0.0899; Figure 2C).

## Discussion

### BDNF blocks LPS-induced effects on GABAergic markers, mainly markers specific of SST interneurons

In this report, we showed that LPS-induced inflammation leads to a significant reduction in the expression of *Sst, Cort*, and *Npy*, key molecular markers of SST interneurons, and that BDNF infusion prior to LPS exposure can prevent these changes. Investigation of other GABAergic markers indicate that the protective effects of BDNF may primarily target SST interneurons, potentially due to their high dependence on BDNF signaling for survival and function (Tripp et al., 2012; Guilloux et al., 2012; Oh et al., 2019).

We showed that LPS-induced inflammation significantly reduced hippocampal BDNF expression, likely due to impaired synthesis and release by pyramidal neurons. Previous studies have reported similar findings, but BDNF levels in those studies were measured at earlier time points (3, 4, and 7 h post-LPS administration; Guan and Fang, 2006; Lapchak et al., 1993; Golia et al., 2019). Our previous study (Rezaei et al., 2024) also demonstrates a reduction in BDNF and GABAergic interneuron markers at 18 h, a time point that extends beyond the early sickness-induced inflammatory response (0–6 h; Dantzer et al., 2008). This later timeframe is more appropriate for investigating molecular mechanisms that may drive longer-term pathophysiological changes, particularly those linked to chronic MDD-like states. Here, the LPS model was not used to assess the well-established early sickness and depression-like behaviors, and instead we focused on cellular changes.

GABAergic interneurons do not synthesize BDNF and therefore rely on its supply from other cells, primarily pyramidal neurons (Cellerino et al., 1998; Gorba and Wahle, 1999). The reduction in BDNF levels suggests a disruption in trophic support to GABAergic interneurons which likely contributes to the downregulation of BDNF-dependent interneuron markers *Sst, Cort,* and *Npy* (Tripp et al., 2012). Importantly, BDNF infusion prior to LPS exposure prevented the reduction in *Bdnf*, supporting the hypothesis that exogenous BDNF can counteract LPS-induced effects, as demonstrated in another study (Wu et al., 2020). To address whether BDNF’s effects reflect general trophic upregulation versus protection under inflammation, we added a BDNF-only baseline group (BDNF/PBS; Supplementary Figures 2, 3). At baseline, BDNF did not change *Sst* or *Cort*, but increased *Npy* mRNA levels and there was no change in BDNF, SST, CORT or NPY protein levels. Thus, the prevention of LPS-induced decreases in *Sst* and *Cort* cannot be explained by baseline upregulation and likely reflects context-dependent preservation of trophic support during inflammation.

### LPS-induced inflammation increases IL-1β and *Gfap* levels, while reducing *Iba1*

The mechanism by which inflammation reduces BDNF levels is not known, but studies show that pro-inflammatory cytokines, in particular IL-1β, decrease BDNF levels (Li et al., 2017; Haddad et al., 2002; Barrientos et al., 2003; Tong et al., 2008). A previous study showed that inactivating astrocytes attenuates LPS-induced effect on BDNF suggesting that astrocyte activation downstream of LPS-induced inflammation may contribute to BDNF depletion (Wang et al., 2019). Consistent with prior studies (Diaz-Castro et al., 2021; Norden et al., 2016), we find that LPS significantly increased IL-1β and *Gfap* levels. Notably, we showed that BDNF infusion did not exhibit any significant anti-inflammatory effects on IL-1β protein levels or *Gfap* mRNA expression. Other studies report anti-inflammatory effects of BDNF on tumor necrosis factor alpha, and interleukin-6 (Jiang et al., 2011; Charlton et al., 2023), as well as heighted inflammation in BDNF heterozygous mice in response to an immune challenge (Parrott et al., 2021). We also observed that *Iba1* expression significantly decreased with LPS, which is opposite to the increase often reported (Hoogland et al., 2015; Chen et al., 2012). However, consistent with our findings, another study found significantly decreased expression of *Iba1* mRNA in brain regions including HPC and cortex (Silverman et al., 2015) and proposed that the suppression of *Iba1* may act as a stop signal to prevent over activation of microglia in response to LPS. In line with their observations, we found elevated IL-1beta levels in the LPS/PBS group despite reduced *Iba1* expression, suggesting that microglia were functionally active to produce cytokines. Another explanation is microglial tolerance, whereby prior inflammation blunts Iba1 reactivity to subsequent LPS exposure. This phenomenon has been demonstrated with repeated LPS injections (Norden et al., 2016; Zhou et al., 2020). In our study, the prior cannula implantation 1 week earlier may have induced a local inflammatory response that had not fully subsided, as reported in other studies (Hayn and Koch, 2015; Holguin et al., 2007). This pre-existing activation could have altered the microglial response to LPS, contributing to the observed reduction in *Iba1* expression.

A limitation of the study is that we did not investigate BDNF-dependent intracellular signaling. BDNF classically binds TrkB and engages MAPK/ERK, PI3K/Akt, and PLC-*γ* pathways that support neuronal survival, dendritic maintenance, and synaptic function (Reichardt, 2006). In inflammatory contexts, LPS binds to Toll-like receptor 4 (TLR4), activating NF-κB, which drives cytokine release and glial reactivity (Dantzer, 2018). Studies indicate that BDNF–TrkB activation counterbalances LPS-induced responses, reducing microglial activation and cytokine production via the TrkB-Erk-CREB signaling pathway (Wu et al., 2020; Charlton et al., 2023). In our study, BDNF pretreatment prevented LPS-induced decreases in SST-related markers while IL-1β and *Gfap* remained elevated. Thus, BDNF’s protective effects likely reflect TrkB-dependent preservation of trophic support and interneuron integrity under inflammatory conditions, rather than a global suppression of the inflammatory cascade. Future experiments in this model should quantify hippocampal p-TrkB, p-ERK, p-Akt. In this study, BDNF ameliorates LPS-induced effects on GABAergic interneurons, despite elevated IL1-beta and *Gfap* levels, suggesting a mechanism that is likely independent of, or downstream from LPS induced microglia and astrocyte activation. This study is not without limitations. We used an acute LPS challenge to model systemic inflammation and test whether hippocampal BDNF prevents LPS-induced reductions in interneuron markers. This approach provides construct validity for immune signaling and causal effects on GABAergic interneurons, but does not capture chronic, low-grade inflammation or the multifactorial dimensions of neuropsychiatric disorders. Accordingly, our findings indicate that BDNF prevents LPS-induced SST interneuron deficits under acute inflammation, rather than implying normalization of a full disease state. We observed discrepancies between mRNA and protein levels for the neuropeptides, particularly BDNF, CORT and NPY. This is consistent with the well-documented phenomenon where mRNA levels do not always fully correlate with protein levels, due to factors such as delays in translation, post-translational modifications, and rapid protein degradation (Liu et al., 2016). As such, the 18-h post-LPS time point may capture changes in transcription, but not fully reflect translated protein levels. Previous studies investigating LPS-induced inflammation have also reported delayed correlation between mRNA and protein levels (Jovanovic et al., 2010). Another limitation of this study is the lack of direct cellular assessments linking molecular changes to functional alterations at the circuit level and histological validation. While we observed changes in GABAergic interneuron markers, future studies should incorporate techniques to determine how these molecular shifts impact neuronal activity, structural alterations and synaptic function.

Finally, the sample size could be expanded to allow for sex-based analyses, and based on our previous findings (Rezaei et al., 2024), we would expect a more robust reduction in some markers in males than in females following LPS exposure.

## Conclusion

This study demonstrates that LPS-induced effects on GABAergic interneurons can be prevented by BDNF, suggesting a protective role for BDNF against inflammation-induced neuronal dysfunction, particularly in SST cells. By linking reduced neuroplasticity, GABAergic deficits, and increased inflammation, the findings provide a mechanistic framework connecting key pathological features of neuropsychiatric disorders.

## Data Availability

The raw data supporting the conclusions of this article will be made available by the authors, without undue reservation.
