# Supplementary material for: Brain-derived neurotrophic factor prevents LPS-induced dysregulation of GABAergic interneuron markers in mouse hippocampus
Source: Front Cell Neurosci. 2025 Sep 25;19:1675003. doi: 10.3389/fncel.2025.1675003 (PMC12507811; doi:10.3389/fncel.2025.1675003)
Supplement: Supplementary file 1 [file Data_Sheet_1.docx]

Supplementary Material

***Vip***

***Cck***

***Crh***

***Pv***

**Brain-derived neurotrophic factor prevents LPS-induced dysregulation of GABAergic interneuron markers in mouse hippocampus**

Sara Rezaei^1,2^ Mounira Banasr^1,2,3^, Thomas D. Prevot^1,2,3^, Yashika Bansal^1^, Erica Vieira^1,3^, Etienne Sibille^1,2,3^

^1^Centre for Addiction and Mental Health (CAMH), 250 College Street, Toronto, ON, M5T 1R8 Canada

^2^Department of Pharmacology and Toxicology, University of Toronto, Medical Sciences Building, 1 King's College Cir Room 4207, Toronto, ON, M5S 1A8, Canada

^3^Department of Psychiatry, University of Toronto, 250 College Street, Toronto, ON, M5T 1R8 Canada

**Supplementary Information Section 1: Materials and Methods**

*Surgery, drug infusion and brain dissection*

Mice were anaesthetized with inhalant isoflurane 3-4% for induction and 1-3% for maintenance. Micro guide was used to implant guide cannula (Protech International INC. Boerne, TX) unilaterally into the hippocampus (HPC) (AP: -1.8mm; ML:+/-0.4mm; DV:-1.8) on either left or right side in a balanced manner across the groups. The micro guide and guide cannula was fixed to the skull with dental cement (catalog no. 1404, Central Dental, Scarborough, Ontario) and two surgical screws (Protech International INC. Boerne, TX). Dummy cannula was inserted into the guide cannula. Three days of post-operative care was performed (metacam 5mg/kg SC every 24 hours and moistened chaw). After one week of recovery, mice were restrained by hand and dummy cannula was removed. Cannula injector (Protech International INC) with 0.5mm projection connected via polyethylene tubing to 5µL Hamilton syringe (Supelco, Bellefonte, PA) was inserted into the guide cannula. Mice were infused with 0.5µL recombinant human BDNF (R&D systems, Biotechne, Minneapolis) at a concentration of 0.5µg/µL (a total 250ng/animal) or sterile PBS at a rate of 0.1ul/min. A total volume of 0.5µL was delivered over 5 minutes. After injections the cannula injector was left in place for an additional 5 minutes to allow diffusion. The dummy cannula was reinserted and thirty minutes following BDNF infusion, ultra-pure lipopolysaccharide (LPS) from E. Coli 0111:B4 strain (InvivoGen, San Diego, CA) was administered intraperitoneally at a dose of 2mg/kg. After 18 hours the mice were euthanized by cervical dislocation. The cortex was peeled and pulled down to dissect the hippocampus (dorsal and ventral) on the side with cannula and was flash-frozen on dry ice.

*Quantitative real-time PCR*

The method for RNA extraction, cDNA synthesis and qPCR has been described in^27^. Briefly, HPC RNA and protein were extracted following the instructions of the Allprep RNA/protein kit (catalogue no. 80404; Qiagen, Hilden, Germany). cDNA was synthesized following the instructions of SuperScript VILO cDNA synthesis kit (cataloge no.11754050; Thermo Fisher Scientific, Waltham, MA). The SsoAdvanced universal SYBR Green supermix (catalogue no.1725275; Bio-Rad, Hercules, CA) and the Mastercycler real time PCR machine (Eppendorf, Hamburg, Germany) was used to run qPCR. The forward and reverse qPCR primer sequences (IDT; Coralville, Iowa) for genes of interest and internal controls are described in supplement table 1. Each qPCR was run in triplicate using 96 well white shell/clear well plates. Three internal controls actin, cyclophilin A, and glyceraldehyde 3 phosphate dehydrogenase were subtracted from the cycle threshold (Ct) values for genes of interest and geomean dCt was calculated. The geomean dCt was used to calculate the relative expression level of each gene using the formula: relative expression level = 2ˆ-dCt*1000. Relative expression level was converted to a percentage by the relative expression level/average expression level of the control PBS/PBS group.

**Supplementary Table 1.** Primers used for quantitative real-time polymerase reaction (qPCR).

| **Gene** | **Forward** | **Reverse** |
| --- | --- | --- |
| *Actb* | CCTAGCACCATGAAGATCAA | GGAAGGTGGACAGTGAGG |
| *Gapdh* | AACTCCCACTCTTCCACCT | CACCACCCTGTTGCTGTA |
| *Ppia (CYCLOPH-A)* | CAAACACAAACGGTTCCCAG | TTCACCTTCCCAAAGACCAC |
| *Bdnf-Cds* | CAGTATTAGCGAGTGGGTCA | CCTTTGGATACCGGGACT |
| *Cck* | GAATAAGTGACCGGGACTACA | GCCCACTACGATGGGTAT |
| *Cort* | CCTTCTCCTCGTGCAAGTA | AGGTCTCGTTGGCATCTC |
| *Npy* | TTAATGAAGGAAAGCACAGAAA | AGATGAGATGAGGGTGGAAA |
| *Pvalb* | ATGAGGTGAAGAAGGTGTTCC | AGCGTCTTTGTTTCTTTAGCAG |
| *Sst* | CAACTCGAACCCAGCAAT | GGTCTGGCTAGGACAACAA |
| *Vip* | GACATCTTGCAGAATCCCTTA | CTGCTGTAATCGCTGGTG |
| *Crh* | AGAAAGGAGAAGAGGAAGAAAACC | CCGCAGCCGCATGTTAG |
| *Gfap* | TGC AGG AGT ACC AGG ATC TAC | GAT CTG GAG GTT GGA GAA AGT C |
| *Aif1 (Iba1)* | GAACCCTCTGATGTGGTCTG | AGGAGGACTGGCTGACTT |

*Enzyme-linked immunosorbent assay (ELISA)*

PierceTM bicinchoninic acid Protein Assay kit (catalog no. 23225, ThermoFisher, Waltham, MA) was used to quantify HPC protein according to manufacturer’s instructions. To measure the protein levels of the neuropeptides ELISA kits for SST (catalog No. MBS701139), NPY (catalog no. MBS701247), CORT (catalog no. MBS2890490) (MyBiosource, San Diego, CA) and BDNF (catalog no. DY248), IL-1β (catalog no. DY401–05) (R&D Systems, Minneapolis, MN) were used according to the manufacturer instructions. For SST, CORT, and NPY the 96-well microplates were pre-coated with capture antibody. Samples and standards were added to the plate and incubated for 2 hours at 37◦C. For SST and CORT the samples were diluted 1:20, and for NPY the dilution factor was 1:10. Liquid was removed without washing and 100µL biotin antibody was added to SST and NPY plates, and 100μL of Detection Reagent A to NPY plate. The plates were incubated for 1hr at 37◦C. The plate was washed 3 times with wash buffer (1x) and 100μL of HRP-avidin was added to SST, NPY plates and 100μL of Detection reagent B to CORT plate. The plates were incubated for 1hr at 37◦C. Following 5 washes, 90μL of TMB substrate solution was added to all plates. SST plate was incubated for 15 minutes, CORT for 5 minutes and NPY for 5 minutes*.* 50μL of stop solution was added to each well and plate was read at 450nm (BioTek™ ELx 800™ Absorbance Reader, Agilent Technologies, Santa Clara, CA). For BDNF and IL-1β, 100μL of diluted capture antibody was added to the 96-well microplate and incubated overnight. The plate was washed with wash buffer (PBS 1X with 0.1%-Triton-X). The plate was blocked by 300μL of reagent diluent (1%-BSA in PBS) for 2hrs, followed by three washes. Diluted samples (1:10) for BDNF and (1:2) for IL-1β and standards were added, followed by overnight incubation at 4◦C. The plate was washed three times, and 100μL of detection antibody was added and incubated for 2hrs. 100μL of working solution streptavidin-HPR was added to each well and the plate was incubated for 20 minutes at room temperature. 50μL of stop solution was added and the plate was read at 450 nm. The average zero standard optical density was subtracted from the standards and samples. The sample values were interpolated by using sigmoidal 4PL to create a standard curve on Prism. The concentration for the samples were multiplied by the dilution factor.


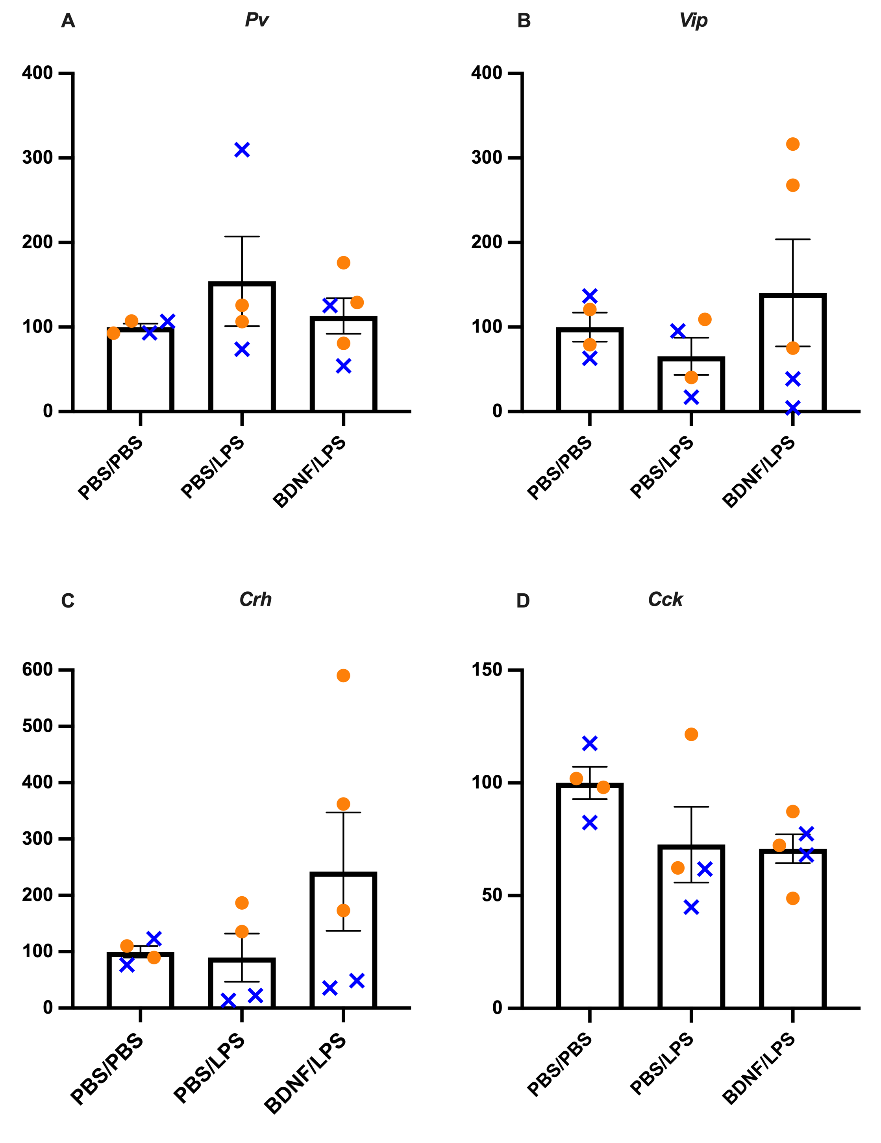
**Supplementary Figure 1.** No significant changes in *Pv, Vip, Crh* or *Cck* gene expression with BDNF or LPS

**% Relative Expression**

(A-D) *Pv, Vip, Crh,* and *Cck* mRNA expression levels. (A) Two by two comparisons showed no significant difference between PBS/LPS group compared to PBS/PBS for *Pv* (P=0.174), *Vip* (P=0.131), *Crh* (P=0.41), or *Cck* in the PBS/LPS group compared to PBS/PBS. Results are expressed as individual mice and mean ± SEM (n = 4-5/group; 50% female). Females are shown as orange circles and males as blue x symbol. *p *<* 0.05, **p *<* 0.01, ***p *<* 0.001 and ****p *<* 0.0001.

**Supplementary Figure 2.** BDNF increased expression of *Npy,* no changes in *Bdnf*, *Sst, Cort, Pv, Vip, Crh,* and *Cck*

**% Relative Expression**

*
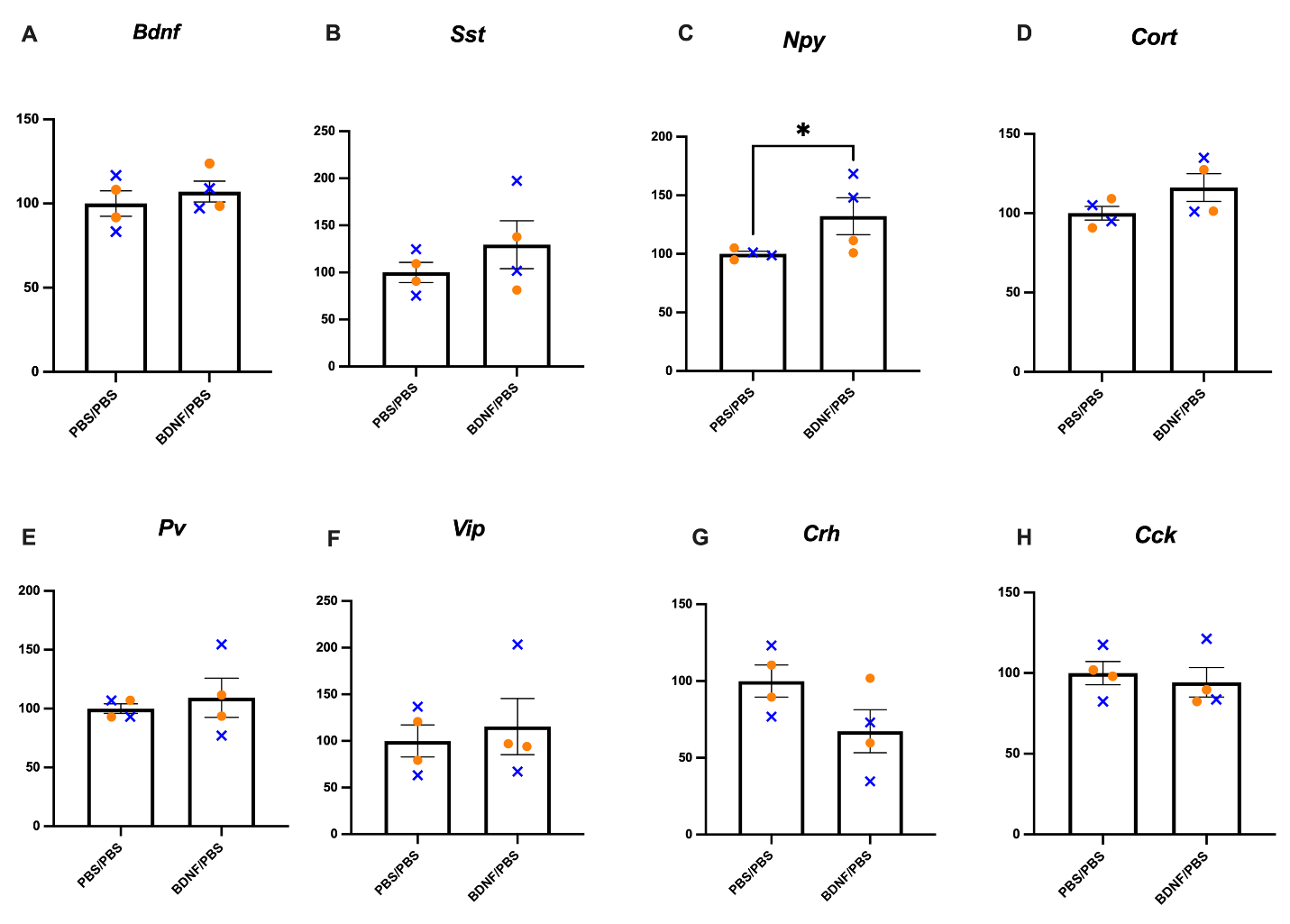
*

(A-G) Effect of BDNF on *Bdnf,* *Sst, Npy, Cort, Pv, Vip, Crh* and *Cck* mRNA expression levels. (A-B) Two by two comparisons showed no significant difference between PBS/PBS group compared to BDNF/PBS for *Bdnf* (P=0.2466) and *Sst* (P=0.1632). (C) For *Npy* there was a significant increase in BDNF/PBS group compared to PBS/PBS (P=0.0445) (D) For *Cort* there was no significant difference between PBS/PBS group compared to BDNF/PBS (P=0.0743). (E-H) There were no significant differences between PBS/PBS group compared to BDNF/PBS for *Pv*, (P=0.3053), *Vip* (P=0.353), *Crh* (P=0.0549) or *Cck* (P=0.3203). Results are expressed as individual mice and mean ± SEM (n = 4-5/group; 50% female). Females are shown as orange circles and males as blue x symbol. *p *<* 0.05, **p *<* 0.01, ***p *<* 0.001 and ****p *<* 0.0001.


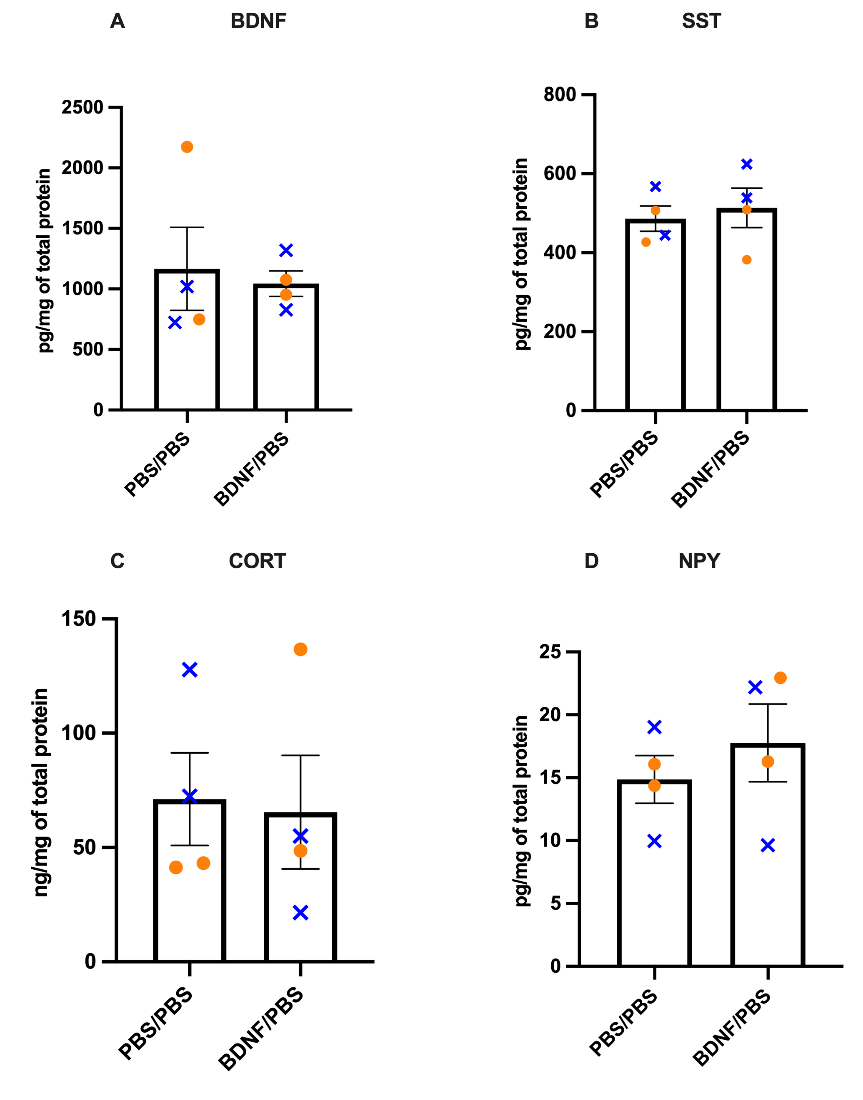
**Supplementary Figure 3.** BDNF had no effect on BDNF, NPY, SST, and CORT protein levels

(A-D) Effect of BDNF on BDNF, SST, NPY, and CORT protein levels. (A-D) Two by two comparisons showed no significant difference between PBS/PBS group compared to BDNF/PBS for BDNF (P=0.3717), SST (P=0.3315), CORT (P=0.4321) and NPY (P=0.2271). Results are expressed as individual mice and mean ± SEM (n = 4-5/group; 50% female). Females are shown as orange circles and males as blue x symbol. *p *<* 0.05, **p *<* 0.01, ***p *<* 0.001 and ****p *<* 0.0001.
